# Antenatal Ultrasonographic Anteroposterior Renal Pelvis Diameter Measurement: Is It a Reliable Way of Defining Fetal Hydronephrosis?

**DOI:** 10.1155/2011/861865

**Published:** 2011-05-17

**Authors:** Alamanda Kfoury Pereira, Zilma Silveira Nogueira Reis, Maria Cândida Ferrarez Bouzada, Eduardo Araújo de Oliveira, Gabriel Osanan, Antônio Carlos Vieira Cabral

**Affiliations:** Fetal Medicine Center, Obstetrics and Gynecology Department, Federal University of Minas Gerais, Alfredo Balena Avenue, 190, Belo Horizonte, CEP 30.130-100, Brazil

## Abstract

*Purpose*. It was to quantify the intraobserver and interobserver variability of the sonographic measurements of renal pelvis and classify hydronephrosis severity. *Methods*. Two ultrasonographers evaluated 17 fetuses from 23 to 39 weeks of gestation. Renal pelvis APD were taken in 50 renal units. For intraobserver error, one of them performed three sequential measurements. The mean and standard deviation from the absolute and percentage differences between measurements were calculated. Bland-Altman plots were used to visually assess the relationship between the precision of repeated measurements. Hydronephrosis was classified as mild (5.0 to 9.9 mm), moderate (10.0 to 14.9 mm), or severe (≥15.0 mm). Interrater agreement were obtained using the Kappa index. *Results*. Absolute intraobserver variation in APD measurements was 5.2 ± 3.5%. Interobserver variation of ultrasonographers was 9.3 ± 9.7%. Neither intraobserver or interobserver error increased with increasing APD size. The overall percentage of agreement with the antenatal hydronephrosis diagnosis was 64%. Cohen's Kappa to hydronephrosis severity was 0.51 (95% CI, 0.33 to 0.69). *Conclusion*. Inter and intraobserver APD measurement errors were low in these group, but the agreement to hydronephrosis diagnosis and classification was fair. We suggest that standard and serial APD measurement can better define and evaluate fetal hydronephrosis.

## 1. Introduction

The advent of routine antenatal ultrasonography has allowed for an appreciation of the true incidence of urological abnormalities and has identified many patients who require reassessment postnatally [1]. In spite of such advances, however, the issue of antenatal hydronephrosis remains a common and challenging problem, with postnatal influences [2, 3].

There have been a number of studies assessing the accuracy of fetal renal pelvic dilatation (RPD) as an indicator of urinary tract anomalies [4–8]. The single most widely used parameter is the anteroposterior diameter (APD) of the renal pelvis, a simple parameter whose application is now widespread in prenatal diagnostics [9]. However, the reproducibility measurement of this parameter has scarcely been investigated. Furthermore, during routine ultrasound examinations, the size of the renal pelvis varies considerably over time [10]. Though the renal collecting system can be influenced by physiological conditions (maternal hydration and degree of bladder filling [11, 12]), the lack of a full technical description and validation of that measurement seems to be a central factor. 

The aim of our investigation was to evaluate the intraobserver and interobserver variability of the APD of the renal pelvis using the standard sonographic method and to verify agreement on antenatal hydronephrosis diagnosis and severity.

## 2. Material and Methods

In a cross-sectional study from August 2007 to December 2008, 19 pregnant women agreed to participate and were followed from 23 to 39 weeks of gestation (mean 33.4 ± 0.6). All of them were referred to the fetal medicine center of the Universidade Federal de Minas Gerais, Brazil, for suspected fetal uropathy. Two cases were excluded because there were other fetal anomalies. The data were collected blindly and prospectively and then analyzed retrospectively. The study was approved by the ethics committee and all participants were informed about the prenatal follow-up protocol, registered by *ETIC 325/05*.

Two trained medical sonography doctors (Pereira AK, Osanan GC) performed all of examinations during the same ultrasonographic appointment. They performed a detailed antenatal renal sonography on 25 appointed US exams from 17 fetuses, resulting in 50 APD measurements. The first observer scanned the right and left APD measurements, three times, in no specific order; the second observer then did the same but only once. The measurement results were blinded to both observers. The maximum interval between each observer exam was one hour.

**Figure 1:**
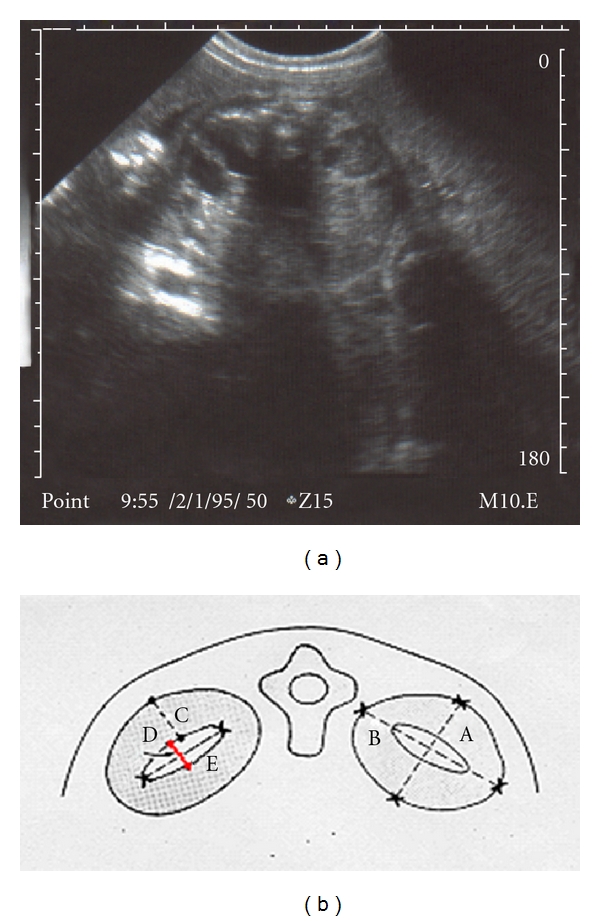
(a) Sonographic transversal view of renal images on either side of the spine, with pelvis dilatation. The arrow shows the anteroposterior diameter. (b) Schematic view of renal images. A—anteroposterior diameter of kidney; B—Transverse diameter of kidney; C—Anteroposterior diameter of renal cortex; D—anteroposterior diameter of renal pelvis (DAP); E—Transverse diameter of renal pelvis.

The maximum APD size was measured using a 5 MHZ linear transabdominal probe, two-dimensional Sonoace-8000 EX Prime, following the standard method: through a transverse slice of the fetal abdomen, a hypoechoic image can be indentified on each side of the spine, corresponding to the kidneys. The renal pelvis appears as an anechoic image on the medial edge of the kidneys. The image obtained is then frozen, and the greatest anterior-posterior distance, parallel to the spine, is measured, as shown in the image and in the schematic drawing (Figure 1). 

All dimensions were measured by 0.1 mm scale. Antenatal hydronephrosis was classified as absent (APD < 5 mm), mild (APD 5.0 to 9.9 mm), moderate (APD 10 to 14.9 mm), and severe (APD ≥ 15 mm) [13, 14].

Interobserver error was calculated by subtracting each APD measured by one observer from the true APD (mean of two observers), for each measurement. The mean and standard deviation of the absolute and percentage differences between APD measurements were calculated by dividing each value by the true APD. The same calculation was performed to determine intraobserver error, when the average of triplicated measurements was obtained. 

It was assumed that the accuracy and precision of ultrasound measurements may depend on bladder filling and APD size. The magnitude of the difference was assessed using paired *t*-tests, considering agreement or lack thereof about bladder status among the observers. Bland-Altman plots were used to provide a visual assessment of whether the difference between the repeat measurements taken by the observers (interobserver error) is dependent on APD size. We plotted the interobserver difference between the two measurements taken by each observer against the average of the two, as well as a line representing the mean difference and ±1.96 SDs (referred to as the 95% limits of agreement).

The overall percentage of agreement with the antenatal hydronephrosis diagnosis was determined and the interrater agreement of prenatal hydronephrosis classification was calculated by Cohen's Kappa with linear weighting and using the 95% limits for all categories. Statistics were calculated using SPSS for Windows (SPSS Inc., Chicago, Ill, USA).

## 3. Results

A dilated fetal renal pelvis (APD ≥ 5.0 mm) was observed in 37 (74%) measurements for observer 1 and in 39 (78%) measurements for observer 2. These results were presented (Table 1). The interobserver variation was 9.3 ± 9.7%. 

The absolute intraobserver variation in 90 APD measurements was 5.2 ± 3.5%. Variance components analysis showed that intraobserver error (difference in repeat measures taken by the same observer) was lower than interobserver error (difference in measurements taken by different observers). 

**Figure 2:**
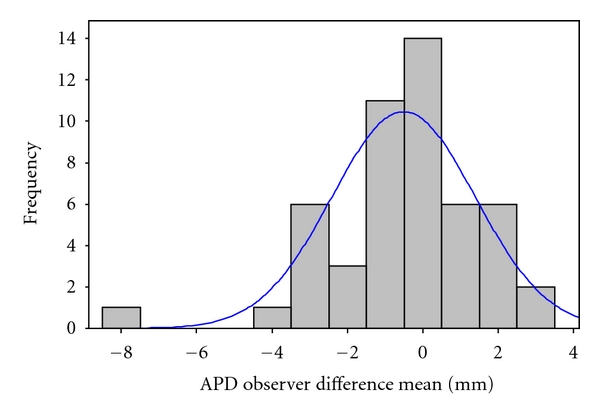
APD: anteroposterior renal pelvis diameter. Anderson Darling test: *P* value  .246.

**Figure 3:**
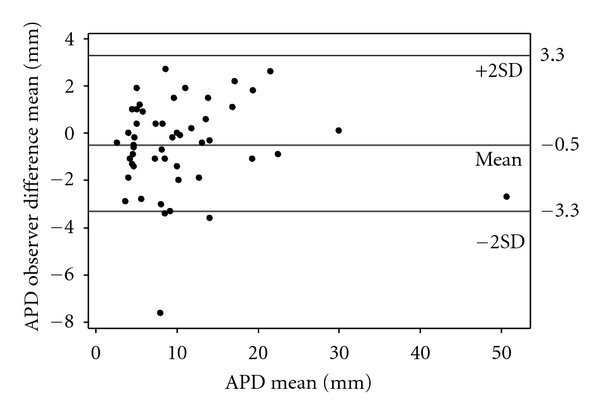
Histogram of interobserver differences for the anteroposterior renal pelvis diameter measurements (by Bland and Altman plot); APD: anteroposterior renal pelvis diameter; SD: standard deviation.

The difference between the repeated measurements taken by the observers (interobserver error) was plotted (Figure 2). The mean and standard deviation of the differences were constant throughout the range of measurements, and these differences are from an approximately Normal distribution (Anderson Darling test: *P* = .246). Additionally, differences did not increase with APD size.  Figure 3 shows Bland and Altman plots for the differences among observers against the APD mean. The limits of agreement (±2SD) are plotted in the figures. Most differences lie between the limits of agreement.

Table 2 shows the interrater reliability for antenatal hydronephrosis diagnosis. Thirty-two antenatal hydronephrosis diagnoses were agreed upon among the ultrasonographers. The overall percentage of agreement was 64%. The percentage of agreement for the mild category was 36.0% (9/25), for the moderate category, it was 56.3% (9/16), and for the severe one, it was 88.9% (8/9). For the absent, mild, moderate, and severe categories, linear weighting of Cohen's Kappa to hydronephrosis severity was 0.51 (95% CI 0.33 to 0.69). 

## 4. Discussion

The ultrasonographic measurement of the APD of the renal pelvis is an antenatal tool that has been widely used for the ante- and postnatal monitoring and conduction of nephrourinary anomalies, without a critical analysis of its reliability.

In this study, absolute intraobserver variation in APD measurements was 5.2 ± 3.5%. Interobserver variation among ultrasonographers was 9.3 ± 9.7%. Neither intraobserver nor interobserver error increased with increasing APD size. The overall percentage of agreement with the antenatal hydronephrosis diagnosis was 64%. Linear weighting of Cohen's Kappa to hydronephrosis severity was 0.51 (95% CI: 0.33 to 0.69).

It can be verified with this data that the measurement of the APD of the renal pelvis should be evaluated in a serial manner, always by the same observer, using the standard sonographic method. It should always be repeated in the postnatal period, ideally after the seventh day [15], in isolated cases of hydronephrosis without other complications. It is important to note that the cutoff levels utilized for the definition and classification of hydronephrosis are variable, and new classification proposals are frequently put forth. We utilized a cutoff level of 5 mm for all gestational ages, with the objective of identifying all the cases that deserved postnatal investigation [5]. 

In this study, there is a broad variability in the gestational age at the time of the diagnosis (24 to 39 weeks). As only one cutoff level was used for the APD, the technical difficulties resulted more from other factors, such as maternal obesity and oligohydramnios, than from the gestational age itself [16].

We admit that some fetal dynamics factors may also have influenced the variability of the APD, that is, the presence of a full bladder and the state of maternal hydration. It was observed that the degree of bladder fullness may influence the dimensions of the fetal renal pelvis. In a study in which only slight dilations were considered, 1/3 were considered highly variable when the degree of bladder fullness was taken into consideration [12]. In another study, dthe anteroposterior diameter of the fetal renal pelvis after hydration did not differ significantly (0.7 versus 0.6 cm *P* < .1) with a full or empty bladder [11]. We agree with these points, and others investigations are necessary to give light to this. 

A factor that was not considered in this investigation and that may interfere in the results, especially regarding the issue of a diagnosis of hydronephrosis and its classification, is the degree of maternal hydration during the exams. In a study involving 13 pregnant women with fetal pielectasia and 13 control women, paired according to gestational age, the diameter of the renal pelvis increased significantly following hydration (0.29 versus 0.46 cm; *P* < .002) in the group with pielectasia and in the control group [17]. On the other hand, no significant difference was found in the renal pelvis diameters both pre- and posthydration in an investigation involving 20 pregnant women [17]. 

Finally, we believe that the standard sonographic measurement methodology can be of fundamental importance. There are few studies that deal with the reproducibility of renal pelvis measurements. One study whose objective was to evaluate the variability in the interpretation of fetal anomalies in second semester ultrasounds, in which 148 ultrasonographers evaluated 46 cases, where renal pielectasia was considered a marker, there was moderate concordance with a Kappa value of 0.51 (CI 95%, 0.50–0.52) [18]. In the present study, despite the concordance having been studied between only two observers, this value was 64%. We carefully standardized the sonographic measurement of the APD and the 0.1 mm scale.

Recently, the reproducibility of the renal pelvis volume, utilizing three-dimensional fetal ultrasound, was investigated, and the intra- and interobserver were considered very good [19]. In another study in which only the reproducibility of fetal bladder volume was studied in 3D (three-dimensional ultrasound fetal urinary bladder volume), an excellent inter- and intraobserver correlation was found [20]. Despite the result of excellent concordance, the technique of calculating the volume using 3D ultrasound requires much more elaborate techniques and equipment and its clinical applicability has yet to be defined. The prenatal diagnosis of uropathies is today the main contributor to the evolution of individual sufferers of this disease. When faced with a diagnosis of pyelocaliceal dilation, postnatal observation should be planned in order to minimize parental anxiety and monitor and intervene in the evolution of the nephrourinary lesions. Because of the extremely elastic nature of the fetal renal system, we suggest that an evaluation of antenatal hydronephrosis via ultrasound be conducted on more than one occasion, using a standardized methodology. Longitudinal, comparative studies with greater technical standardization may come to elucidate the dynamic nature of hydronephrosis and better define the prognostic criteria.

## Figures and Tables

**Table 1:** The characteristics of anteroposterior renal pelvis diameter (APD) measurements.

	*n*	Mean ± SD	Range (mm)	APD < 5 mm	APD 5.0–9.9 mm	APD 10–14.9 mm	APD ≥ 15 mm
Observer 1	50	10.2 ± 8.2	2.1–49.3	13 (26.0%)	18 (36.0%)	11 (22.0%)	8 (16.0%)
Observer 2	50	10.7 ± 8.2	2.8–52.0	11 (22.0%)	14 (28.0%)	14 (28.0%)	9 (18.0%)
Mean APD	50	10.5 ± 8.2	2.6–50.7	12 (16.0%)	18 (36.0%)	12 (24.0%)	8 (16.0%)

APD: *anteroposterior renal pelvis diameter*, SD: standard deviation.

**Table 2:** The interrater reliability for antenatal hydronephrosis diagnosis of 50 anteroposterior renal pelvis diameter measurements.

		Hydronephrosis (observer 1)				Total
		Absent	Mild	Moderate	Severe	
Hydronephrosis (observer 2)	Absent	6	5	0	0	11
	Mild	6	9	1	0	16
	Moderate	1	4	9	0	14
	Severe	0	0	1	8	9

	Total	13	18	11	8	50

Linear weighting Cohen's Kappa: 0.51 (0.33 to 0.69).
